# Arabic version of the Extended Nordic Musculoskeletal Questionnaire, cross-cultural adaptation and psychometric testing

**DOI:** 10.1186/s13018-023-04161-1

**Published:** 2023-09-11

**Authors:** Hamad S. Al Amer, Ahmad A. Alharbi

**Affiliations:** Department of Physical Therapy, Faculty of Applied Medical Sciences, University of Tabuk, 71491 Tabuk, Saudi Arabia

**Keywords:** Arabic, Musculoskeletal pain, Nordic Musculoskeletal Questionnaire, Adaptation, Reliability, Validity

## Abstract

**Background:**

The Extended Nordic Musculoskeletal Questionnaire (NMQ-E) had never been adapted into Arabic. We culturally adapted the NMQ-E to Arabic in five stages and investigated its psychometric properties.

**Methods:**

After translating and cross-culturally adapting the NMQ-E into Arabic, through forward translation, translation synthesis, backward translation, expert committee review, and pilot testing, the psychometric properties were investigated. The construct validity was assessed by having the participants completing the Arabic version of the NMQ-E with four Arabic scales that measured musculoskeletal pain in all regions covered in the NMQ-E. Participants’ baseline records were also used to evaluate internal consistency (Cronbach’s *α*). Seven days later, participants completed the Arabic NMQ-E again to evaluate its test–retest reliability employing intraclass correlation coefficient (ICC) and standard error of measurement (SEM) for the age questions, and Cohen’s kappa coefficient (*κ*), the proportion of observed agreement (*P*_o_), the proportion of positive agreement (*P*_pos_), and the proportion of negative agreement (*P*_neg_) for the dichotomous items.

**Results:**

A total of 127 participants, 70 males and 57 females with mean age 32.5 ± 12.2 years, were included. Significant differences were found between participants with and without musculoskeletal pain in the corresponding scales, signifying the content validity of the questionnaire. Cronbach’s *α* for both the prevalence and consequences pain sections combined ranged from 0.30 to 1.00. The test–retest reliability of the age questions was excellent, with ICC values ranging between 0.995 and 1.00. The SEM was 0.77 for the neck region and 0 for the rest of the regions. The prevalence questions demonstrated almost perfect agreement, with κ values ranging between 0.82 and 1.00, the *P*_o_ between 0.94 and 1.00, the *P*_pos_ between 0.80 and 1.00, and the *P*_neg_ between 0.93 and 1.00. The consequences of pain questions showed moderate-to-perfect agreement, with κ values ranging between 0.57 and 1.00, between 0.83 and 1.00 for the *P*_o_, between 0.50 and 1.00 for the *P*_pos_, and between 0.86 and 1.00 for the *P*_neg_.

**Conclusions:**

The results suggest that the Arabic NMQ-E is a valid and reliable tool that can be used to screen Arab adults for the prevalence and consequences of musculoskeletal pain.

**Supplementary Information:**

The online version contains supplementary material available at 10.1186/s13018-023-04161-1.

## Background

Musculoskeletal conditions (aches, pain, or discomfort) are prevalent issues with direct impacts on people of all ages, from young adults to the elderly, and are known to have significant effects on quality of life [1–3]. Musculoskeletal disorders represent a public health challenge of serious concern because they often lead to disability and absenteeism among workers [4]. Consequently, this issue has a considerable impact on personal, social, and economic circumstances [1].

Reliable data collection is a fundamental aspect of epidemiological investigations of musculoskeletal pain (MSP) [5]. Hence, it is necessary to employ valid and reliable epidemiological instruments to effectively survey musculoskeletal disorders and facilitate the development of appropriate therapeutic interventions [6].

The Nordic Musculoskeletal Questionnaire (NMQ) was presented by Kuorinka et al. [7] to measure MSP and its consequences among the general population [8, 9], and more commonly, among occupational populations [10–12]. Around 20 years later, Dawson et al. [5] developed an Extended version of the NMQ (NMQ-E), aiming to produce larger and more useful datasets concerning the prevalence and consequences of MSP. They concluded that the NMQ-E provided reliable data on the prevalence and repercussions of MSP. Pugh et al. [13] devised and created an online version of the NMQ-E, which they stated to be reliable and valid for assessing MSP.

Results and conclusions drawn from self-reported measures such as the NMQ-E are essential for clinical evaluation and research. The NMQ-E is a practical instrument that provides reliable and valid data on the onset, incidence, and consequences of MSP in the neck, shoulders, upper back, elbows, wrists and hands, lower back, hips and thighs, and feet in occupational and general populations. The cross-cultural adaptation of such questionnaire facilitates the comparison of research findings on MSP across diverse cultural contexts, thereby promoting knowledge exchange within the global scientific community and reducing associated expenses and time for establishing new measure [14–16]. In Fact, the NMQ-E has been culturally adapted into Persian [17], Turkish [18], and Hebrew [6]; however, to our knowledge, the NMQ-E has never been translated and adapted into Arabic—the official language of 23 countries. Hence, the translation and cultural adaptation of the NMQ-E to Arabic were our study objectives. Additionally, this study investigated the psychometric characteristics of the translated questionnaire.

## Material and methods

The study was conducted in two stages. The NMQ-E was translated and cross-culturally adapted into Arabic in the first stage, and its psychometric properties were evaluated in the second one.

### Translation and cross-cultural adaptation

Permission to translate and cross-culturally adapt the NMQ-E to Arabic was obtained from Dawson via email [5]. The NMQ-E was translated into five phases based on the advice given by Beaton et al. [19]. In the first stage (forward translation), two native Arabic speakers fluent in English translated the questionnaire from English to Arabic. The first translator was a physiotherapist with 20 years of experience and familiarity with the NMQ-E concept, whereas the second translator was a computer engineer with neither a medical background nor awareness of the NMQ-E concept. In the second stage (translation synthesis), the translators synthesized the two Arabic versions into approved version. In the third phase (backward translation), two native English speakers fluent in Arabic translated the Arabic version into English. The translators lacked a medical background and had no access to the original version of the NMQ-E. During the fourth phase (expert committee), an expert committee comprising the four translators, a physiotherapist, and a methodologist reviewed and analyzed any discrepancies or inconsistencies found in the previous stages of the translation process. The team also compared the reverse-translated versions with the original NMQ-E. A field-testing version of the pre-final Arabic version (Arabic NMQ-E) was produced after a discussion and implementation of all necessary modifications to ensure clarity and suitability for the general Arab public. The only notable modification was changing the sentence “Please answer questions from left to right” to be read, in Arabic, as “Please answer questions from right to left,” as well as reversing the order of items to be suitable for the Arabic version. In the fifth stage (content validity assessment and pilot testing), 15 health professionals (five family medicine physicians, five physical therapists, and five nurses) were invited to complete the survey and rank the clarity and relevance of each item on a rating scale of 1 to 4: 1 = unclear/irrelevant, 2 = somewhat clear/somewhat relevant, 3 = clear/relevant, and 4 = very clear/very relevant [20]. This was performed using the pre-final version to determine content validity. Next, the pre-final version was completed by 30 participants, including teaching staff, administrative staff, and laboratory technicians from the physical therapy and nursing departments at the University of Tabuk, to evaluate the comprehensibility, clarity, and suitability for each questionnaire item. The respondents did not report any significant difficulties and demonstrated reading and comprehension abilities for all items. Finally, the Arabic NMQ-E was created and has become suitable for psychometric testing.

### Psychometric testing

#### Participants

Using a convenience sample, adults aged 18 years or older from Tabuk City, Saudi Arabia, and the surrounding local communities were invited to participate. The invitation to participate was via word-of-mouth and personal communication. After signing the informed consent, participants’ demographic data and past surgical and medical history were collected. Within the previous six months, participants with a history of severe chronic systemic, psychological, neurological, or significant musculoskeletal conditions (e.g., rheumatoid arthritis, fibromyalgia, or traumatic injury) or surgeries in the musculoskeletal system) were excluded.

#### Procedure

The qualified participants completed the new translation in two sessions: baseline and follow-up assessments. To our knowledge, there is no single validated Arabic scale that evaluates MSP in the body regions within the NMQ-E. Therefore, participants completed the Arabic versions of the Neck Disability Index (NDI) [21]; Oswestry Disability Index (ODI) [22]; shortened Disabilities of Arm, Shoulder, and Hand questionnaire (Quick-DASH) [23]; and the Lower Extremity Functional Scale (LEFS) [24] to evaluate the construct (convergent) validity of the translated NMQ-E in Session 1. Seven days later, during Session 2 (the follow-up assessment), participants completed a seven-level global change scale to identify any noteworthy changes in MSP since baseline. On the aforementioned global change scale, participants ranked the degree that their MSP had shifted over the past seven days as *completely gone, much better, better, a little better, about the same, a little worse,* or *much worse*. Participants who opted for options “about the same” or “a little better” or “a little worse” were cases showing stability and thus, re-completed the Arabic NMQ-E.

### Measures

#### NMQ-E

The NMQ-E is a self-administered or face-to-face questionnaire. The questionnaire provides reliable data on the onset and prevalence of MSP in nine body regions, including the neck, shoulders, upper back, elbows, wrists/hands, lower back, hips/thighs, and ankles/feet, over four distinct time periods (lifetime, annual, monthly, and present). Moreover, the NMQ-E evaluates the impact of ache, pain, or discomfort encountered in the abovementioned body regions on work and daily activities, in addition to the need for medical care owing to MSP (hospitalization, medication, and visiting healthcare providers) [5].

#### NDI

The NDI is a valid questionnaire used to evaluate the impact of neck pain on the functional status of patients in clinical practice and research [25]. It has been translated and culturally adapted into Arabic and was shown to be reliable and valid [21]. The NDI includes 10 items on pain intensity, personal care, lifting, reading, headache, concentration, work, driving, sleeping, and recreation [25]. Each item covers six statements ranging from 0 (*no disability*) to 5 (*maximal disability*). The maximum possible score for disability is 50, which is the sum of the scores for each item. The NDI score is usually normalized to 100 and reported as a percentage [25].

#### ODI

The ODI is another valid, reliable, and sensitive questionnaire used in patients with low back pain (LBP) to evaluate the level of functional disability [26]. The Arabic version of the ODI is valid and reliable [22]. It encompasses 10 items that address pain intensity and the impact of LBP on function (personal care, lifting, walking, sitting, standing, sleeping, sex life, social life, and traveling). Each item has six statements ranging from 0 (*no disability*) to 5 (*maximal disability*). Patients are required to select the statement most suitable for describing their status/condition. Once the total sum is calculated, it is divided by the total possible score (i.e., 50) to obtain the disability score. The resulting total is multiplied by 100 to reveal the percentage of the patient’s disability, where 0% represents (no disability) and 100% serves as (the most severe disability) [26].

#### Quick-DASH

The Quick-DASH is a shorter version of the 30-item DASH, which has comparable psychometric properties to the full version [27]. Similarly, in addition to being reliable, the Arabic version of Quick-DASH is a valid self-reported outcome measure in patients with various upper-extremity impairments [23]. It contains 11 items assessing the severity of symptoms (pain and tingling) and the level of difficulty in performing a variety of functional and social activities involving the upper extremities, in addition to work and sleep [28]. Each item of the Quick-DASH comprises five response options, ranging from 1 (*no difficulty or no symptoms*) to 5 (*unable to engage in activity or very severe symptoms*). The score of disability is calculated by adding and averaging the values for all completed responses to get a result of 5 or less; this is followed by transforming this value to a hundred. To this extent, the higher the result, the greater the level of severity in terms of disability [29].

#### LEFS

Another self-administered questionnaire used to evaluate the activity confines of patients with lower extremity musculoskeletal disorders is the LEFS [30]. Like other scales, this tool was translated, culturally adapted, and validated in Arabic language [24]. It comprises 20 region-specific items, and each item’s score ranges from 0 (*very difficult or unable to perform the activity*) to 4 (*no difficulties of performing the activity*), with a maximum possible score of 80. The LEFS can also be expressed as a percentage; the lower the percentage, the greater the disability/limitation [30].

### Statistical analysis

The approach selected for data analysis included evaluation of the Arabic NMQ-E for validity and reliability. Table 1 shows the a priori hypotheses through which all the obtained values were assessed. Statistical tests were performed using IBM SPSS Statistics for Windows (version 25.0; Armonk, NY, USA).

**Table 1:** A priori hypotheses for evaluating the psychometric characteristics of the Arabic version of the Extended Nordic Musculoskeletal Questionnaire

*Reliability*	
Internal consistency	Cronbach’s *α* = 0.70–0.95 [35]
Test–retest	Continuous items: ICC ≥ 0.70 [35] SEM ≤ 0–3.8 [5, 13, 17]
	Dichotomous items: *κ* ≥ 0.70 [35] *P*_o_ ≥ 0.64 [5, 6, 13] *P*_pos_ ≥ 0.30 [5, 6, 13] *P*_neg_ ≥ 0.46 [5, 6, 13]
*Validity*	
Content validity	I-CVI ≥ 0.78 [36] S-CVI/Ave ≥ 0.90 [36] S-CVI/UA ≥ 0.80 [36]
Construct validity	Respondents that reported musculoskeletal pain on the Arabic NMQ-E will score significantly higher than those without pain on the NDI (neck pain), Quick-DASH (shoulders, elbows, and wrists/hands pain), ODI (upper and lower back pain), and significantly lower in the LEFS (hips/thighs, knees, and ankles/feet pain)

*ICC* intraclass correlation coefficient, *SEM* standard error of measurement, *κ* Cohen’s kappa coefficient, *P*_o_ proportion of observed agreement, *P*_pos_ proportion of positive agreement, *P*_neg_ proportion of negative agreement, *I-CVI* item-level content validity index, *S-CVI/Ave* average scale-level content validity index, *S-CVI/UA* universal agreement, *NMQ-E* extended Nordic Musculoskeletal Questionnaire, *NDI* Neck Disability Index, *Quick-DASH* shortened Disabilities of Arm, Shoulder, and Hand questionnaire, *ODI* Oswestry Disability Index, *LEFS* Lower Extremity Functional Scale

### Validity

#### Content validity

The item-level content validity index (I-CVI), average scale-level content validity index (S-CVI/Ave), and universal agreement (S-CVI/UA) were used to analyze the content validity. To determine the I-CVI, the sum of experts who rated an item as three or four was divided by the total number of experts; to determine the S-CVI/Ave, all I-CVIs were added and divided by the total number of items; and to determine the S-CVI/UA, the number of I-CVIs equal to 1.00 was divided by the total number of items [20].

#### Construct validity

The construct validity of the Arabic NMQ-E was evaluated by testing the difference between respondents with and without complaints in each region using the average score of the relevant questionnaire. We hypothesized that respondents who reported MSP on the Arabic NMQ-E will score significantly higher than those without pain on the NDI (for neck pain), Quick-DASH (for shoulders, elbows, and wrists/hands pain), ODI (for upper and LBP), and significantly lower in the LEFS (for hips/thighs, knees, and ankles/feet pain). Owing to unequal and/or small group sizes in some instances, Mann–Whitney U tests were conducted to examine significance with an alpha level of 0.05.

### Reliability

Reliability testing included internal consistency and test–retest reliability. Cronbach’s α was computed at baseline to estimate the internal consistency for each region for the questions pertaining to prevalence (four questions), consequences of pain (six questions), and for both sections combined (10 questions). Items with zero variance were excluded from analysis. The test–retest reliability of the dichotomous items between baseline and a week later was assessed by determining Cohen’s kappa coefficient (*κ*), the proportion of observed agreement (*P*_o_), the proportion of positive agreement (*P*_pos_), and the proportion of negative agreement (*P*_neg_). The proportion of agreement was interpreted as follows: < 0, less than chance agreement; 0.01–0.20, slight agreement; 0.21–0.40, fair agreement; 0.41–0.60, moderate agreement; 0.61–0.80, substantial agreement; and 0.81–0.99, almost perfect agreement [31]. The intraclass correlation coefficient (ICC) was used to measure the reliability of questions pertaining to age. Values of the Cronbach’s *α* and ICC were interpreted as follows: < 0.50, poor; 0.50–0.75, moderate; 0.75–0.90, good; and > 0.90, excellent [32, 33]. Further, the standard error of measurement (SEM) was calculated using the following formulas: $SEM=SD\sqrt{1-ICC}$ (SD is the standard deviation) [34]. For test–retest reliability, only the responses of participants who were determined as stable on the follow-up assessment were used.

## Results

### Characteristics of participants

A total of 127 participants were included in the assessment of the psychometric characteristics of the Arabic NMQ-E. Out of those participants, 116 were categorized as stable and included in the test–retest analysis. Thus, the number of participants recommended for psychometric testing was met [35]. The demographic characteristics of the participants are presented in Table 2. Table 3 shows the MSP prevalence rates for lifetime, year, and month periods. The lifetime prevalence of MSP ranges from 5.5% in the elbows to 33.9% in the lower back region. The highest annual prevalence was found in the lower back (21.3%), followed by the neck (18.9%). Similarly, the prevalence rates during the last month for the lower back and neck regions were the highest at 11.8%. At the time of the study, the region that most participants complained of was the lower back (7.9%), and none reported any problems with their elbows.

**Table 2:** Demographic characteristics of the participants (*n* = 127)

Characteristic	*n*	%
Sex		
Male	70	55.1
Female	57	44.9
Marital status		
Single	65	51.2
Married	58	45.7
Divorced	4	3.1
Education level		
Middle school	4	3.1
High school	37	29.1
Diploma	8	6.3
University	71	55.9
Postgraduate education	7	5.5
Employment status		
Employed	48	37.8
Unemployed	36	28.3
Retired	8	6.3
Student	35	27.6
Age mean ± SD (years)	32.5 ± 12.2	
Weight mean ± SD (kg)	71.1 ± 16.9	
Height mean ± SD (m)	1.70 ± 0.1	

*SD* standard deviation

**Table 3:** Prevalence of musculoskeletal pain by period and by region (*n* = 127)

Region	Lifetime (%)	Year (%)	Month (%)	Point (%)
Neck	31.5	18.9	11.8	4.7
Shoulders	22	13.4	6.3	2.4
Upper back	11.8	6.3	3.9	3.1
Elbows	5.5	2.4	1.6	0
Wrists/hands	10.2	6.3	5.5	3.1
Low back	33.9	21.3	11.8	7.9
Hips/Thighs	13.4	7.9	4.7	3.1
Knees	18.1	13.4	8.7	3.1
Ankles/feet	15.7	8.7	3.9	3.9

### Validity

#### Content validity

Table 4 summarizes the results of the content validity test for the Arabic NMQ-E. For clarity, all items scored higher than the recommended I-CVI of 0.70, ranging between 0.87 and 1.00. Thus, all items were regarded as clear. The clarity of S-CVI/Ave was 0.98, indicating excellent content validity. The clarity of S-CVI/UA was 0.73. Similar results were obtained for relevancy. The I-CVI ranged between 0.93 and 1.00, indicating that every item is relevant. The relevancy S-CVI/Ave was 0.98, which also indicates excellent content validity. The S-CVI/UA equaled 0.64. All calculated values of the content validity of the Arabic NMQ-E were consistent with our hypotheses, except for S-CVI/UA for both clarity and relevancy, which fell slightly below the suggested levels [36].

**Table 4:** Content validity assessment of the Arabic version of the Extended Nordic Musculoskeletal Questionnaire

Variable	Clarity component	Relevance component
Number of Items with I-CVI ≥ 0.70	11	11
Number of Items with I-CVI < 0.70	0	0
Minimum–Maximum I-CVI	0.87–1.00	0.93–1.00
S-CVI/Ave	0.98	0.98
S-CVI/UA	0.73	0.64

Values were calculated using content validity analysis

*I-CVI* item-level content validity index, *S-CVI/Ave* average scale-level content validity index, *S-CVI/UA* universal agreement

#### Construct validity

The means and standard deviations of participants’ scores on the NDI, ODI, Quick-DASH, and LEFS at baseline are listed in Table 5. Regarding lifetime prevalence, there were significant differences between participants with and without MSP in all nine body regions. Those who reported MSP scored significantly higher than those without pain on the NDI, Quick-DASH, and ODI, and significantly lower on the LEFS. Similar results were found in the neck, knees, and ankles/feet for point prevalence, as well as in the shoulders, upper back, knees, and ankles/feet for monthly prevalence. For annual prevalence, the only significant difference was found in the neck region. These results, particularly for lifetime prevalence, supported our hypothesis regarding the construct validity of the Arabic NMQ-E.

**Table 5:** Differences in the level of disability/pain between participants with and without pain

Time prevalence		Lifetime					Year				
Region	Index	Yes		No		*p*-value	Yes		No		*p*-value
		*n*	*M* (SD)	*n*	*M* (SD)		*n*	*M* (SD)	*n*	*M* (SD)	
Neck	NDI	40	15.6 (9.6)	87	3.2 (7.3)	< 0.001*	24	18.4 (9.0)	16	11.4 (9.3)	0.023*
Shoulders	Quick-DASH	28	15.6 (12.8)	99	5.5 (11.9)	< 0.001*	17	14.8 (10.7)	11	17.0 (16.0)	0.759
Upper back	ODI	15	14.7 (14.3)	112	5.9 (10.7)	0.004*	8	20.8 (16.0)	7	7.7 (8.6)	0.114
Elbows	Quick-DASH	7	22.7 (25.4)	120	6.9 (11.2)	0.043*	3	20.4 (6.8)	4	24.4 (35.4)	0.721
Wrists/ Hands	Quick-DASH	13	25.2 (23.0)	114	5.8 (9.3)	< 0.001*	8	23.3 (18.5)	5	28.2 (31.2)	1.00
Low back	ODI	43	16.0 (14.2)	84	2.3 (5.9)	< 0.001*	27	18.1 (14.7)	16	12.3 (13.0)	0.124
Hips/ Thighs	LEFS	17	78.8 (18.4)	110	91.0 (14.9)	0.001*	10	71.0 (19.2)	7	90.0 (10.0)	0.051
Knees	LEFS	23	76.9 (19.7)	104	92.1 (13.5)	< 0.001*	17	74.5 (20.5)	6	83.8 (16.8)	0.360
Ankles/ Feet	LEFS	20	82.6 (20.1)	107	90.6 (14.7)	0.043*	11	74.0 (23.2)	9	93.1 (8.1)	0.09

Time prevalence		Month					Point				
Region	Index	Yes		No		*p*-value	Yes		No		*p*-value
		*n*	*M* (SD)	*n*	*M* (SD)		*n*	*M* (SD)	*n*	*M* (SD)	
Neck	NDI	15	20.1 (7.5)	10	15.5 (11.0)	0.188	6	24.3 (3.2)	19	16.3 (9.2)	0.044*
Shoulders	Quick-DASH	8	22.4 (6.8)	9	8.1 (9.0)	0.005*	3	16.6 (5.3)	14	14.4 (11.6)	0.899
Upper back	ODI	5	31.0 (9.4)	3	4.0 (7.0)	0.024*	4	17.5 (16.8)	4	24.0 (17.0)	0.559
Elbows	Quick-DASH	2	20.4 (9.6)	1	20.5 (0)	1.00	0	(−)^a^	3	20.4 (6.8)	(−)^a^
Wrists/ Hands	Quick-DASH	7	22.5 (19.8)	1	29.5 (0)	0.513	4	21.6 (15.8)	4	25.1 (23.3)	0.773
Low back	ODI	15	22.0 (12.2)	12	13.3 (16.7)	0.035	10	21.1 (14.0)	17	16.4 (15.3)	0.364
Hips/ Thighs	LEFS	6	71.7 (20.9)	4	70.0 (19.4)	0.831	4	71.6 (15.6)	6	70.6 (22.7)	0.831
Knees	LEFS	11	65.6 (16.4)	6	90.8 (17.7)	0.016*	4	52.5 (18.3)	13	81.3 (16.4)	0.023*
Ankles/ Feet	LEFS	5	54.3 (15.9)	6	90.4 (12.6)	0.006*	5	54.5 (16.1)	6	90.2 (13.0)	0.013*

*p*-values were calculated with Mann–Whitney *U* test

*NDI* Neck Disability Index, *Quick-DASH* shortened Disabilities of Arm, Shoulder, and Hand questionnaire, *ODI* Oswestry Disability Index, *LEFS* Lower Extremity Functional Scale, *M* mean, *SD* standard deviation

*Significant difference at *α* = 0.05

^a^Value cannot be calculated

### Reliability

#### Internal consistency

The results of the internal consistency analysis are presented in Table 6. For questions pertaining to prevalence, the Cronbach’s *α* values ranged from 0.30 to 0.82 for shoulders and low back regions, respectively. Regarding the questions on the consequences of pain, the internal consistency ranged between 0.60 and 1.00 for ankle/feet and elbows regions, respectively. Overall, the internal consistency of values of both prevalence and consequences of pain sections combined were within the hypothesized range for elbows, wrists/hands, lower back, hips/thighs and knees (0.77–0.92), and just below the recommended value for neck, shoulders, and upper back regions (0.63–0.69). The lowest Cronbach’s α value was calculated for the ankles/feet items (0.49).

**Table 6:** Internal consistency analysis of the Arabic version of the Extended Nordic Musculoskeletal Questionnaire (*n* = 127)

Body region	Subscale	No. of items	Cronbach’s *α*
Neck	Prevalence questions^a^	3	0.54
	Consequences of pain questions^a^	5	0.72
	All questions	8	0.63
Shoulders	Prevalence questions^b^	2	0.30
	Consequences of pain questions	6	0.69
	All questions	8	0.63
Upper back	Prevalence questions^b^	2	0.70
	Consequences of pain questions	6	0.77
	All questions	8	0.69
Elbows	Prevalence questions^d^	1	-
	Consequences of pain questions^c^	3	1.00
	All questions	4	0.92
Wrists/Hands	Prevalence questions^b^	2	0.52
	Consequences of pain questions	6	0.92
	All questions	8	0.84
Low back	Prevalence questions^b^	2	0.69
	Consequences of pain questions	6	0.82
	All questions	8	0.77
Hips/Thighs	Prevalence questions^b^	2	0.80
	Consequences of pain questions	6	0.81
	All questions	8	0.77
Knees	Prevalence questions^b^	2	0.58
	Consequences of pain questions	6	0.81
	All questions	8	0.80
Ankles/Feet	Prevalence questions^b^	2	0.78
	Consequences of pain questions	6	0.60
	All questions	8	0.49

^a^One item with zero variance was excluded from the analysis

^b^Two items with zero variance were excluded from the analysis

^c^Three items with zero variance were excluded from the analysis

^d^Cronbach’s *α* cannot be calculated because too many items with zero variance were excluded from the analysis

#### Test–retest reliability

The test–retest reliability of the age-of-onset questions was excellent, with ICC values ranging between 0.995 and 1.00. The SEM was 0.77 for the neck region and 0 for the rest of the regions (Table 7). In addition, the prevalence questions demonstrated almost perfect agreement. The κ values ranged between 0.82 and 1.00, the *P*_o_ between 0.94 and 1.00, the *P*_pos_ between 0.80 and 1.00, and the *P*_neg_ between 0.93 and 1.00. However, the test–retest reliability values of the consequences of pain questions showed some variability (Table 8). The κ values ranged between 0.57 and 1.00, between 0.83 and 1.00 for the *P*_o_, between 0.50 and 1.00 for the *P*_pos_, and between 0.86 and 1.00 for the *P*_neg_, thus indicating moderate-to-perfect agreement of the consequences of pain questions. Except for the κ value for the annual prevention from work question for the upper back, and the annual sick leave question for the shoulders, all test–retest values calculated for the Arabic NMQ-E confirm our hypotheses stated in Table 1.

**Table 7:** Test–retest reliability of the age of onset and prevalence questions of the Arabic version of the Extended Nordic Musculoskeletal Questionnaire

Body region	*n*	Age at onset		Lifetime prevalence				Year prevalence			
		ICC (95% CI)	SEM	*κ*	*P*_o_	*P*_pos_	*P*_neg_	*κ*	*P*_o_	*P*_pos_	*P*_neg_
Neck	116	0.995 (0.989–0.998)	0.77	0.98	0.99	1.00	0.99	1.00	1.00	1.00	1.00
Shoulders	119	1.00 (−)	0	0.97	0.99	1.00	0.99	1.00	1.00	1.00	1.00
Upper back	121	1.00 (−)	0	0.94	0.99	1.00	0.99	1.00	1.00	1.00	1.00
Elbows	126	1.00 (−)	0	1.00	1.00	1.00	1.00	1.00	1.00	1.00	1.00
Wrists/Hands	123	1.00 (−)	0	1.00	1.00	1.00	1.00	1.00	1.00	1.00	1.00
Low back	114	1.00 (−)	0	0.98	0.99	1.00	0.99	1.00	1.00	1.00	1.00
Hips/Thighs	123	1.00 (−)	0	0.96	0.99	1.00	0.99	1.00	1.00	1.00	1.00
Knees	122	1.00 (−)	0	0.97	0.99	1.00	0.99	1.00	1.00	1.00	1.00
Ankles/Feet	122	1.00 (−)	0	0.96	0.99	1.00	0.99	1.00	1.00	1.00	1.00
Mean	–	0.999	–	0.97	0.99	1.00	0.99	1.00	1.00	1.00	1.00

Body region	*n*	Month prevalence				Point prevalence					
		*κ*	*P*_o_	*P*_pos_	*P*_neg_	*κ*	*P*_o_	*P*_pos_	*P*_neg_		
Neck	116	1.00	1.00	1.00	1.00	0.82	0.94	1.00	0.93		
Shoulders	119	1.00	1.00	1.00	1.00	1.00	1.00	1.00	1.00		
Upper back	121	1.00	1.00	1.00	1.00	1.00	1.00	1.00	1.00		
Elbows	126	1.00	1.00	1.00	1.00	*	1.00	*	1.00		
Wrists/Hands	123	*	1.00	1.00	*	1.00	1.00	1.00	1.00		
Low back	114	1.00	1.00	1.00	1.00	0.89	0.95	0.88	1.00		
Hips/Thighs	123	1.00	1.00	1.00	1.00	1.00	1.00	1.00	1.00		
Knees	122	1.00	1.00	1.00	1.00	0.85	0.94	0.80	1.00		
Ankles/Feet	122	1.00	1.00	1.00	1.00	1.00	1.00	1.00	1.00		
Mean	–	1.00	1.00	1.00	1.00	0.95	0.98	0.96	0.99		

Test–retest reliability values were calculated using agreement analysis and ICC and SEM for the age-of-onset questions

*ICC* intraclass correlation coefficient, *CI* confidence interval, *SEM* standard error of measurement, *κ* Cohen’s kappa coefficient, *P*_o_ proportion of observed agreement, *P*_pos_ proportion of positive agreement, *P*_neg_ proportion of negative agreement

*Value cannot be calculated

**Table 8:** Test–retest reliability of the consequences of pain questions of the Arabic version of the Extended Nordic Musculoskeletal Questionnaire

Body region	*n*	Lifetime hospitalization				Lifetime changed jobs or duties				Annual prevention of normal work			
		*κ*	*P*_o_	*P*_pos_	*P*_neg_	*κ*	*P*_o_	*P*_pos_	*P*_neg_	*κ*	*P*_o_	*P*_pos_	*P*_neg_
Neck	116	*	1.00	*	1.00	1.00	1.00	1.00	1.00	1.00	1.00	1.00	1.00
Shoulders	119	*	1.00	*	1.00	0.83	0.95	1.00	0.94	1.00	1.00	1.00	1.00
Upper back	121	*	1.00	*	1.00	1.00	1.00	1.00	1.00	0.57	0.83	0.80	1.00
Elbows	126	1.00	1.00	1.00	1.00	1.00	1.00	1.00	1.00	1.00	1.00	1.00	1.00
Wrists/Hands	123	1.00	1.00	1.00	1.00	0.73	0.89	1.00	0.86	1.00	1.00	1.00	1.00
Low back	114	1.00	1.00	1.00	1.00	1.00	1.00	1.00	1.00	0.89	0.95	0.92	1.00
Hips/Thighs	123	0.81	0.93	0.75	1.00	1.00	1.00	1.00	1.00	1.00	1.00	1.00	1.00
Knees	122	1.00	1.00	1.00	1.00	1.00	1.00	1.00	1.00	0.88	0.94	0.89	1.00
Ankles/Feet	122	0.76	0.93	1.00	0.92	0.76	0.93	1.00	0.92	0.72	0.86	0.75	1.00
Mean	–	0.93	0.98	0.96	0.99	0.92	0.97	1.00	0.96	0.92	0.96	0.93	1.00

Body region	*n*	Annual visit to health professional				Annual medication				Annual sick leave			
		*κ*	*P*_o_	*P*_pos_	*P*_neg_	*κ*	*P*_o_	*P*_pos_	*P*_neg_	*κ*	*P*_o_	*P*_pos_	*P*_neg_
Neck	116	1.00	1.00	1.00	1.00	1.00	1.00	1.00	1.00	1.00	1.00	1.00	1.00
Shoulders	119	1.00	1.00	1.00	1.00	1.00	1.00	1.00	1.00	0.63	0.92	0.50	1.00
Upper back	121	1.00	1.00	1.00	1.00	1.00	1.00	1.00	1.00	1.00	1.00	1.00	1.00
Elbows	126	1.00	1.00	1.00	1.00	*	1.00	*	1.00	1.00	1.00	1.00	1.00
Wrists/Hands	123	1.00	1.00	1.00	1.00	1.00	1.00	1.00	1.00	1.00	1.00	1.00	1.00
Low back	114	0.90	0.95	0.91	1.00	1.00	1.00	1.00	1.00	1.00	1.00	1.00	1.00
Hips/Thighs	123	1.00	1.00	1.00	1.00	1.00	1.00	1.00	1.00	1.00	1.00	1.00	1.00
Knees	122	0.87	0.94	1.00	0.86	1.00	1.00	1.00	1.00	1.00	1.00	1.00	1.00
Ankles/Feet	122	0.72	0.86	0.75	1.00	1.00	1.00	1.00	1.00	0.70	0.86	0.67	1.00
Mean	–	0.92	0.96	0.94	0.98	1.00	1.00	1.00	1.00	0.95	0.98	0.95	1.00

Test–retest reliability values were calculated using agreement analysis

*κ* Cohen’s kappa coefficient, *P*_o_ proportion of observed agreement, *P*_pos_ proportion of positive agreement, *P*_neg_ proportion of negative agreement

*Value cannot be calculated

## Discussion

The NMQ-E provides dependable data on the onset, prevalence, and outcomes of MSP in nine body regions and is a valid and useful tool for use in occupational and general populations [5, 8–12]. However, the cross-cultural validity and reliability of this questionnaire must be determined before it can be used in diverse contexts. In this study, and in accordance with published guidelines [19], the NMQ-E was successfully translated, cross-culturally adapted, and psychometrically tested for use in Arab population.

The English version of the NMQ-E was translated and adapted into Arabic through a systematic method of forward translation, version synthesis, back-translation, and build-up by subject matter experts. Throughout the translation, cultural adaptation, and pilot stages, no major changes to the items or responses were necessary, and the final version of the Arabic NMQ-E demonstrated a high degree of comprehension. The only noteworthy modification was changing the sentence “Please answer questions from left to right” to be read, in Arabic, as “Please answer questions from right to left.” The reason is that Arabic language is written from right to left. Similarly, the order of items was also reversed to be from right to left in the Arabic version.

The results demonstrated that the Arabic NMQ-E is a valid tool for obtaining data on the onset, prevalence, and outcomes of MSP in nine body regions in adults. In particular, the Arabic NMQ-E validity was demonstrated in two ways.

First, 15 multidisciplinary medical field experts rated the clarity and relevance of the Arabic NMQ-E as part of the content validity assessment. This was performed by calculating the CVI for each component in accordance with the recommendations of Zamanzadeh and colleague [20]. The Arabic NMQ-E had excellent content validity, according to the calculated CVI values (Table 4).

Additionally, none of the 30 adults who answered the pre-final Arabic version of the NMQ-E experienced significant difficulties in responding to the questionnaire. In approximately five minutes, participants could read, understand, and complete all the questions. Further, the expert committee translated and adapted the Arabic questionnaire into the Modern Standard Arabic [37] to strengthen the applicability of the currently adopted Arabic version in all Arabic-speaking nations.

Second, to examine the construct validity, we hypothesized that there are significant differences between participants who reported MSP and those who did not. The results supported our hypothesis regarding the construct validity of the Arabic NMQ-E, mainly for the lifetime prevalence rate. Specifically, participants who reported MSP on the Arabic NMQ-E scored significantly higher than those without pain on the NDI (for neck pain), Quick-DASH (for shoulders, elbows, and wrists/hands pain), ODI (for upper and LBP), and significantly lower in the LEFS (for hips/thighs, knees, and ankles/feet pain). These findings coincide with previous validation studies of the NMQ-E regardless of the validation approach used. For example, Alaca and colleagues [18] validated the Turkish version of the NMQ-E by testing difference in the Turkish Cornell Musculoskeletal Discomfort questionnaire [38] between the participants with and without MSP in NMQ-E and found significant differences between the two groups. Pugh et al. [13] examined the validity of the modified English NMQ-E utilizing exploratory factor analysis and concluded that it is a homogenous measure of MSP.

Cronbach’s *α*, ICC, SEM, *κ*, *P*_o_, *P*_pos_, and *P*_neg_ were used to determine the internal consistency and test–retest reliability of the Arabic NMQ-E. The results of the reliability analysis suggest that the Arabic NMQ-E has adequate reliability. The internal consistency of values of both prevalence and consequences of pain sections combined was within the range suggested by Terwee and colleagues [35] for elbows, wrists, hands, lower back, and knees (0.77–0.92) and just below the recommended value for neck, shoulders, and upper back regions (0.63–0.69). In comparison with previous validation studies of the NMQ-E, Cronbach’s alpha for the Arabic version was slightly higher for some anatomical regions and lower for others. For instance, the modified English NMQ-E [13] showed higher values of Cronbach’s α than the Arabic-NMQ-E for neck, shoulders, upper back, low back, hips/thighs, knees and ankles/feet of 0.86, 0.89, 0.90, 0.88, 0.89, 0.92, respectively, and lower values for elbows and wrists/hands of 0.81 and 0.92, respectively. The overall internal consistency for the Turkish version [18] was found 0.78 for the Turkish version [18], and 0.03–0.74 for the Hebrew version [6].

All calculated test–retest values for the Arabic NMQ-E supported the predefined hypotheses presented in Table 1. With ICC values ranging from 0.995 to 1.00, the test–retest reliability of the age-at-onset questions was determined to be excellent, implying stability over time. Terwee et al. [35] recommended an ICC of at least 0.70. Similar to previous studies, this study revealed an excellent ICC for the age item, at 0.85–1.00 for the Hebrew version [6], 0.88 for the Turkish version [18], > 0.7 for the Persian version [17], and 0.87–1.00 for the original NMQ-E [5]. Further, the SEM was 0.77 for the neck region and the rest of the regions were 0. In comparison with previous reports, this range of SEM value is smaller than the range calculated for the Persian version (0.56–1.76) [17], the original NMQ-E (0.49–1.84) [5], and the modified English NMQ-E (0–3.83) [13]. The low SEM score in our study indicates that the Arabic NMQ-E has high absolute reliability [34].

Using Kappa statistics, the test–retest reliability of the Arabic NMQ-E for prevalence questions was high, with agreement indices (κ, *P*_o_, the *P*_pos_, and *P*_neg_) ranging between values between 0.82 and 1.00. This result indicates an almost perfect agreement and is relatively higher than the values reported in previous studies for the original NMQ-E (0.35–1.00) [5], the modified English NMQ-E (0.30–1.00) [13], and the Hebrew version (0.19–1.00) [6]. However, the consequences of the pain questions demonstrated moderate-to-perfect agreement, with agreement indices ranging from 0.50 to 1.00. These values, as well as those calculated in other reports [5, 6, 13], highlight some variability in the test–retest reliability of the consequences of pain questions. The original NMQ-E showed agreement indices ranging between 0.10 and 1.00 [5], between 0.30 and 1.00 for the modified English NMQ-E [13], and between 0.46 and 1.00 for the Hebrew version [6]. This variability among studies could be explained by the test–retest intervals, statistics, and samples used. For example, the sample used for the original NMQ-E were nursing students, and the time interval for test–retest was 24 h [5]. However, the sample used for the Hebrew and Turkish versions of the NMQ-E was physiotherapists and physiotherapy and rehabilitation students with a 7-day time interval for test–retest reliability [6, 18]. Despite the use of various test–retest periods, data, and samples, the adopted versions of the NMQ-E presented in this study showed adequate test–retest reliability. When studying the test–retest reliability of self-reported measures, a memory effect could occur if a brief time interval is used between sessions. Alternatively, a change in status could occur over a longer timeframe. Seven days was selected as a compromise between shorter and longer intervals between baseline and retesting [5, 39].

This study had some limitations. First of all, participants were screened for eligibility using self-reported questions rather than examining them with valid and reliable tools or reviewing their past medical records. Second, all the questionnaires were administered in the same order, which could have resulted in an ordering effect. Third, we did not evaluate operational qualities, such as the time required to complete the questionnaire. Fourth, most of the participants included in the pilot study were highly educated (at least high school graduates). Although the translation of the Arabic NMQ-E was completed to be understandable by a 12-year-old (roughly a sixth-grade literacy level) [19], including participants with primary school education could have made the pilot study more relevant in terms of comprehensibility evaluation of the questionnaire. Additionally, we did not evaluate the degree of understanding of each item during the pilot study using a quantitative method, such as calculating the Misunderstanding Index. Although each participant was interviewed and asked about the clarity of the questionnaire items and responses, utilizing such measure could be more useful at that stage of the adaptation process. Finally, we recommend additional research to be conducted to examine the viability of the Arabic version in other Arab populations, as well as the potential to use it as a web-based assessment method.

## Conclusion

The Arabic NMQ-E generated valid and reliable data on the onset, prevalence, and consequences of MSP in Arab adults. The availability of such tools is beneficial for epidemiological studies, screening for MSP, increasing awareness of musculoskeletal disorders, and developing programs aimed at preventing and improving musculoskeletal disorders.

### Supplementary Information

**Additional file 1.** Raw Data.

## Data Availability

All data generated or analyzed during this study are included in this published article [Raw Data.xlsx].

## Abbreviations

NMQ-E: The Extended Nordic Musculoskeletal Questionnaire
MSP: Musculoskeletal pain
NMQ: The Nordic Musculoskeletal Questionnaire
NDI: Neck Disability Index
ODI: Oswestry Disability Index
Quick-DASH: Shortened Disabilities of Arm, Shoulder, and Hand questionnaire
LEFS: Lower Extremity Functional Scale
LBP: Low back pain
I-CVI: Item-level content validity index
S-CVI/Ave: Average scale-level content validity index
S-CVI/UA: Universal agreement
*κ*: Cohen’s kappa coefficient
*P*_o_: Proportion of observed agreement
*P*_pos_: Proportion of positive agreement
*P*_neg_: Proportion of negative agreement
ICC: Intraclass correlation coefficient
SEM: Standard error of measurement
SD: Standard deviation
